# First Lines in Midwifery

**Published:** 1892-06

**Authors:** 


*First Lines in Midwifery.* By G. ERNEST HERMAN, M.D. Pp. 191. London: CASSELL & CO. LIMITED. 1891.

This book, which is intended for midwives as well as students, contains a great deal of very useful information, but not of a strictly bedside character, as it gives elementary details, anatomical and otherwise, which are of very little use to the midwife, and are not full enough for the student. Although there is much that is good in the work and some little that is new and original, it scarcely carries out its professed purpose. It would be more correctly entitled "A Guide for Medical Students and Midwives preparing for Examination in Midwifery." It would be a capital book with which to refresh the memory a few days before the examination.

